# Long-Term Survival in a Patient with Multiple Brain Metastases from Small-Cell Lung Cancer Treated with Gamma Knife Radiosurgery on Four Occasions: A Case Report

**DOI:** 10.1155/2012/276189

**Published:** 2012-10-04

**Authors:** Ameer L. Elaimy, Sudheer R. Thumma, Andrew F. Lamm, Alexander R. Mackay, Wayne T. Lamoreaux, Robert K. Fairbanks, John J. Demakas, Barton S. Cooke, Christopher M. Lee

**Affiliations:** ^1^Department of Neurosurgery, Gamma Knife of Spokane and Cancer Care Northwest, 601 S. Sherman, Spokane, WA 99202, USA; ^2^Department of Radiation Oncology, Gamma Knife of Spokane and Cancer Care Northwest, 601 S. Sherman, Spokane, WA 99202, USA

## Abstract

Brain metastases are the most common cancerous neoplasm in the brain. The treatment of these lesions is challenging and often includes a multimodality management approach with whole-brain radiation therapy, stereotactic radiosurgery, and neurosurgery options. Although advances in biomedical imaging technologies and the treatment of extracranial cancer have led to the overall increase in the survival of brain metastases patients, the finding that select patients survive several years remains puzzling. For this reason, we present the case of a 70-year-old patient who was diagnosed with multiple brain metastases from small-cell lung cancer five years ago and is currently alive following treatment with chemotherapy for the primary cancer and whole-brain radiation therapy and Gamma Knife radiosurgery on four separate occasions for the neurological cancer. Since the diagnosis of brain metastases five years ago, the patient's primary cancer has remained controlled. Furthermore, multiple repeat GKRS procedures provided this patient with high levels of local tumor control, which in combination with a stable primary cancer led to an extended period of survival and a highly functional life. Further analysis and clinical research will be valuable in assessing the durability of multiple GKRS for brain metastases patients who experience long-term survival.

## 1. Introduction

The treatment of brain metastases remains a uniformly challenging issue for neurosurgeons and oncologists due to the numerous factors that must be taken into account when prescribing treatment regimens. Today, it is well known that clinical outcomes are dependent on Karnofsky performance status (KPS), age, control of primary cancer, and the presence of extracranial metastases [1]. In addition, treatments must be further tailored to the individual patient based on the number, location, and size of brain metastases they present with at the time of diagnosis. The combination of these variables makes treating these cancerous lesions a very complex issue that requires experience and extensive knowledge of the evolving body of world literature.

Although several randomized controlled trials have been published analyzing the efficacy of whole-brain radiation therapy (WBRT), stereotactic radiosurgery (SRS), neurosurgery, and combinations of the three modalities in the management of patients with brain metastases, many questions remain unanswered because the Phase III clinical evidence is often limited by studies with poor patient accrual and conflicting results [2–5]. On average, patients diagnosed with brain metastases treated with steroid therapy alone survive one to two months [2]. The eradication of tumor cells in the brain undergoing rapid mitosis with WBRT can extend the average survival of select patient from four to seven months [2]. Furthermore, in several published reports, local treatment of these neoplasms with surgical resection or SRS alone or in combination with WBRT has proven to be a more effective management approach for select patients when compared to WBRT alone [2–5]. 

Since tumor recurrence is frequently observed following the initial treatment of patients with brain metastases, the efficacy of adjuvant therapies is a subject that has become more prevalent in the literature. A recent randomized controlled trial reported that patients with one to three brain metastases treated with salvage WBRT following tumor recurrence, after surgery or SRS, experienced superior levels of local and distant tumor control when compared to patients who were monitored with observation only [6]. However, the increased levels of tumor control in this patient subset did not result in longer periods of survival [6]. Due to its high local tumor control rate, SRS is also commonly used to treat recurrent brain metastases usually in cases where a new lesion arises and previously treated lesions are stable. 

Despite the fact that the management of patients with brain metastases has improved and moderately longer periods of survival have been reported, the finding that select patients survive several years remains puzzling. For this reason, we present the case of a 70-year-old patient who was diagnosed with multiple brain metastases from small-cell lung cancer (SCLC) five years ago and is currently alive following treatment with chemotherapy for the SCLC and WBRT and Gamma Knife radiosurgery (GKRS) on four separate occasions for recurrent brain disease. 

## 2. Case Presentation

The patient in this case is a 70-year-old female diagnosed with multiple brain metastases from primary small-cell carcinoma of the lung. She reported a significant smoking history (100 packs per year) with COPD and later developed a small-cell carcinoma of the upper lobe of the left lung. Following her diagnosis, she underwent a left upper lobe lobectomy and tolerated the surgery well. Spread to the mediastinal and hilar nodes was not observed at that time. However, tumor evidence was seen in the soft tissues surrounding the bronchial resection margin, and a single involved lymph node was noted in the pathology report of the lobectomy resection. Imaging studies performed at that time did not show evidence of the disease elsewhere in the body. 

Although SCLC is known to be a radiosensitive tumor histology, the patient was not felt to be a candidate for radiation therapy due to her limited pulmonary reserve. The patient was prescribed chemotherapy with Carboplatin and VP-16. The chemotherapy was given in 4 cycles within a period of three months, and the patient responded well to the treatment. A CT scan of the chest performed three months after the resection showed no evidence of tumor recurrence. The patient had no complaints at that time except for some mild shortness of breath.

Approximately five months later, the patient presented with complaints of headaches and blurred vision. She also reported nausea without vomiting. An MRI scan revealed multiple brain metastases (three lesions). The largest of them was in the left cerebellar hemisphere (1.4 cm^3^). Other masses were noted in the left frontal lobe (0.56 cm^3^) and right putamen (0.49 cm^3^). The patient was then prescribed Decadron and her neurological symptoms significantly improved. 

After discussing the available treatment options with a radiation oncologist, WBRT was prescribed to the patient for a period of three weeks. The total radiation dose prescribed was 37.5 Gy in 15 fractions. An MRI performed one month later revealed continued postcontrast enhancement of the masses when compared to the previous MRI. The option of being treated with GKRS was then presented, and risks and benefits were discussed with the patient. The patient consented to GKRS and underwent the treatment without reporting any complications. The Gamma Knife dose prescribed for each lesion was 16 Gy to the 50% isodose line. 

An MRI performed two months following GKRS revealed that the left cerebellar lesion and left frontal lesion decreased in size and the right putamen lesion exhibited stable changes. No new lesions were observed at that time. Follow-up MRIs were performed at two-to-three month intervals for a period of 24 months and were stable without any new foci. During this period, the patient presented with episodes of transient ischemic attacks. An MRI performed three months following the initial attacks showed the presence of two new lesions in the right caudate region (0.21 cm^3^) and right centrum region (0.054 cm^3^). The patient consented to repeat GKRS and underwent the procedure without reporting any complications. The previously irradiated left cerebellar lesion (1.3 cm^3^) and left frontal lesion (0.49 cm^3^) were also treated. The Gamma Knife dose prescribed was again 16 Gy to the 50% isodose line for each lesion. The second Gamma Knife procedure was performed approximately 27 months following the first procedure. 

Again, the patient was followed both clinically and with serial MRI scans at two-to-three month intervals and was found to have radiographic documentation of a new brain metastasis directly medial to the previously irradiated left cerebellar lesion (0.13 cm^3^). The other lesions were reported to have stable changes. The patient was fully informed about the risks and benefits of being treated with a third Gamma Knife procedure. The patient consented to the treatment and was prescribed a dose of 16 Gy to the 55% isodose line. The third Gamma Knife procedure was performed approximately 16 months following the second procedure. The patient tolerated the treatment well and did not report any complications. 

Follow-up appointments with both a medical oncologist and radiation oncologist revealed a stable primary cancer and minor muscle weakness from steroid use. A serial MRI performed approximately three months following the third Gamma Knife procedure revealed a new brain metastasis within the right insular cortex (0.061 cm^3^). The other treated lesions were reported to have stable changes. The patient consented to a fourth Gamma Knife procedure and was prescribed a dose of 16 Gy to the 50% isodose line. The fourth Gamma Knife procedure was performed approximately three months following the third procedure. 

Further follow-up appointments and serial MRI scans revealed stability in both her primary cancer and her neurological cancer. No new brain lesions have been reported. The patient was diagnosed with brain metastases 60 months ago and is still alive and experiencing a good quality of life despite some minor chronic neurological symptoms. She continues to be monitored closely. 

## 3. Discussion

 SRS using the Gamma Knife is frequently used as an initial and adjuvant treatment for patients with brain metastases from lung cancer. At the Lars Leksell Gama Knife Center of the University of Virginia, Pan et al. [7] treated 171 patients with non-small-cell lung cancer (NSCLC) and 20 patients with SCLC with GKRS (total of 424 lesions). As expected, patients who were <65 years of age with a KPS ≥ 70 exhibited superior levels of survival when compared to the other evaluated patients. The NSCLC and SCLC treatment arms did not statistically differ in terms of median survival. Similar to the patient in our case, multiple GKRS procedures were associated with longer periods of survival. It is likely that this increase in survival is a result of the high local tumor control that GKRS is known to provide. In a retrospective cohort study, Jawahar et al. [8] treated 44 patients with brain metastases from carcinoma of the lung. In clinical analysis, it was reported that the ability to achieve local tumor control following GKRS and a stable primary cancer was associated with superior levels of survival (*P* < 0.01) when compared to the survival of the other studied patients. 

 Other clinical studies have also found GKRS to be an effective treatment for patients with multiple brain metastases from SCLC following initial treatment with WBRT. Specifically, in an analysis of 51 patients with brain metastases from SCLC who experienced WBRT failure, Harris et al. [9] reported adjuvant GKRS to provide a one-year and two-year local control rate of 57% and 34%, respectively. Although the median survival of this patient subset was 5.9 months, the authors concluded that a significant predictor of overall survival was the status of the patient's extracranial disease. Since the primary cancer of the patient in our case remained stable for multiple years, this factor could be a reason why she experienced an extended period of survival with a strong quality of life. 

 Varlotto et al. [10] evaluated tumor control and radiation-related toxicity in 137 brain metastases patients (208 lesions) who had survived at least one year following treatment with GKRS. The median period of followup was 18 months, and 38 patients (28%) were diagnosed with multiple brain metastases. The reported one-year and five-year local control rates were 89.6 ± 2.1% and 62.8 ± 6.9%, respectively. In addition, 23 ± 3.6% of patients experienced distant intracranial relapse at one year after radiosurgery and 67.1 ± 8.7% of patients experienced distant intracranial relapse at five years after radiosurgery. These results suggest that the outcomes of GKRS for brain metastases are correlated with the long-term local control rates that select patients experience following the treatment. This improved local control can lead to an extended period of survival in some patients. 

 A retrospective review of 51 brain metastases patients who experienced long-term survival (≥2 years) at the University of Minnesota was performed by Hall et al. [11]. Of the 51 patients, 16 (31%) of them were diagnosed with multiple brain metastases. It was reported that, at two years after the initial diagnosis of brain metastases, patients with metastases from ovarian carcinoma experienced the greatest period of survival. Interestingly, at two years after the initial brain metastasis diagnosis, patients with a SCLC primary experienced the shortest period of survival. In addition, there were no patients with brain metastases from SCLC who survived five years or more. Although this finding is at odds with the period of survival experienced by the SCLC patient in this case, cumulative literature suggests that the survival of brain metastases patients is dependent on other factors in addition to primary tumor histology (e.g., treatment regimen, KPS, age, control of primary cancer, etc.) [2–5]. 

## 4. Conclusion

This case report highlights the long-term survival of a patient with multiple brain metastases from SCLC who has been treated with chemotherapy for the primary cancer and WBRT and GKRS on four occasions for the neurological cancer. Since the diagnosis of brain metastases five years ago, the patient's primary cancer has remained controlled. Furthermore, multiple repeat GKRS procedures provided this patient with high levels of local tumor control, which in combination with a stable primary cancer led to an extended period of survival and a highly functional life. Further analysis and clinical research will be valuable in assessing the durability of multiple GKRS for brain metastases patients who experience long-term survival. 
